# The regulatory effect of 6-TG on lncRNA–miRNA–mRNA ceRNA network in triple-negative breast cancer cell line

**DOI:** 10.1042/BSR20203890

**Published:** 2021-02-03

**Authors:** Daoyu Zhang, Xinglan An, Hao Yu, Ziyi Li

**Affiliations:** 1 Key Laboratory of Organ Regeneration and Transplantation of Ministry of Education, First Hospital, Jilin University, Changchun 130021, China; 2 College of Animal Science, Jilin University, Changchun, Jilin 130062, China

**Keywords:** ceRNA, Thioguanine (6-TG), Triple-negative breast cancer (TNBC)

## Abstract

Breast cancer is one of the most prevalent and recurring cancer types that leads to deaths in women. Triple-negative breast cancer (TNBC) is difficult to treat due to the lack of therapeutic targets. Many studies have focused on identifying drugs for use as alternative treatments for breast cancer. Thioguanine (6-TG) exerts antitumor effects in cancer. Increasing evidence has demonstrated that competitive endogenous ribonucleic acids (ceRNAs) are involved in cancer processes. However, the mechanism by which 6-TG regulates lncRNA–miRNA–mRNAs has not been elucidated. We evaluated the antitumor effect of 6-TG in MDA-MB-231 cells and comprehensively analyzed the RNA-Seq data of MDA-MB-231 cells treated with 6-TG. Our results showed that most tumor pathways were blocked by 6-TG. The hub genes were *FN1, FLNA, FLNB, VCL, GSN, MYH10, ACTN4, KDR* and *EREG*, and they were all down-regulated after 6-TG treatment. The coexpression network consisted of 18 microRNAs (miRNAs), 9 long noncoding RNAs (lncRNAs) and 20 mRNAs. *Hsa-mir-16-5p* and *Hsa-mir-335-5p* targeted the greatest number of mRNAs in the network. These molecules could bind to *PAX8-AS1* and eliminate the inhibition of target mRNA expression. We showed that *PAX8-AS1* is the main lncRNA affected by 6-TG and that *PAX8-AS1* regulates the hub genes in tumor pathways by competitively binding with *miR-16-5p* and *miR-335-5p*.

## Introduction

Breast cancer is one of the common causes of deaths in women. Among all diagnosed cases of invasive breast cancer, approximately 15–20% are triple-negative breast cancer (TNBC) [1], a cancer type without estrogen receptor (ER), progesterone receptor (PR) and human epidermal growth factor receptor 2 (HER-2). TNBCs, more often, occur in young women and have a worse prognosis than other breast cancer subtypes. They are difficult to treat due to the lack of targets for molecularly guided therapies [2]. To date, chemotherapy is still the only systematic treatment for TNBC, and screening FDA-certified drugs used to treat other diseases to identify treatments for TNBC is one of the tentative alternative treatment strategies [3].

Thioguanine (6-TG) is a classic leukemia therapy drug with potential for cancer therapy [4]. 6-TG acts as a DNA methylation regulator in acute lymphoblastic leukemia cells. It reactivates epigenetically silenced genes by facilitating proteasome-mediated degradation of *DNMT1* [5]. Inki et al. [6] reported that 6-TG had a significant antitumor effect against pancreatic ductal adenocarcinoma with low levels of thiopurine methyltransferase. Furthermore, Tao et al. [7] suggested that 6-TG induced G_2_-M arrest and cell death by DNA mismatch repair mediated in human colorectal carcinoma. Our previous studies have shown that 6-TG can induce FAS-mediated exogenous apoptosis and p21-dependent G_2_/M arrest by restoring TP53 activity in MCF-7 breast cancer cells [8].

Intensive research in the past two decades has uncovered the presence and importance of noncoding RNAs (ncRNAs), which include microRNAs (miRNAs) and long ncRNAs (lncRNAs) [9]. LncRNAs are emerging as new players in gene regulation and are associated with the development of cancers [10]. An increasing number of studies have indicated that lncRNAs are involved in cell growth, apoptosis, cell migration and invasiveness as well as cancer cell stemness in breast cancer. It has been reported that lncRNA *BCRT1* is overexpressed in breast cancer and promotes breast cancer metastasis by targeting *miR-1303* [11]. Wu et al. [12] reported that lncRNA *NKILA* suppresses TGF-β-induced epithelial–mesenchymal transition by blocking NF-KB signaling in breast cancer. Therefore, the theory of competitive endogenous ribonucleic acid (ceRNA) has emerged, which states that lncRNAs can competitively combine with the miRNA response element (MRE) and inhibit the negative regulation of target mRNAs by miRNAs. The ceRNA network has been confirmed in many diseases. However, the role of ceRNA in the regulation of breast cancer remains to be further studied.

In our study, we found that 6-TG plays an antitumor role in TNBC cells (MDA-MB-231 cells). To elucidate the interactive mechanism of lncRNAs and the lncRNA-mediated regulatory network, we performed an integrative analysis to identify differentially expressed mRNAs (DEmRNAs) and lncRNAs (DElncRNAs) of TNBC cells after treatment with 6-TG and identified a ceRNA network that can down-regulate core genes that dominate the tumorigenesis process. The present study contributes to the exploration of the ceRNA regulatory mechanism of 6-TG and provides valuable insight for further functional research.

## Materials and methods

### Cells and chemicals

The human breast cancer cell line MDA-MB-231 was purchased from the Cell Bank of the Institute of Basic Medical Sciences, Chinese Academy of Medical Sciences. Cells were cultured in RPMI 1640 medium (C11875500, Gibco, U.S.A.) supplemented with 10% fetal calf serum, 100 IU/ml penicillin and 100 μg/ml streptomycin (HyClone, U.S.A.). The cells were cultured in a humid environment with 5% CO_2_ at 37°C. The MDA-MB-231 cell line has been authenticated using STR profiling within the last 3 years. All experiments were performed with mycoplasma-free cells.

6-TG was purchased from Selleck Chemicals (S1774, Selleck, U.S.A.). Dimethyl sulfoxide (DMSO) was purchased from Sigma–Aldrich (D2650, Sigma, U.S.A.).

### Proliferation assay

Cells were treated with different concentrations of 6-TG and then subjected to the Cell Counting Kit-8 (CCK-8) assay according to the manufacturer’s protocol (Y6002, Everbright®Inc). Briefly, 6.5 × 10^3^ cells were seeded per well in a 96-well plate and treated with 6-TG at 0.5, 1, 2, 4 and 8 μM and DMSO. Thereafter, CCK-8 solution was added, and the absorbance was measured using a microplate reader (Bio-Rad, U.S.A.) at 450 nm.

### RNA preparation and RNA-seq

Total RNA was extracted with TRIzol (15596026, Invitrogen, U.S.A.) reagent following the manufacturer’s instructions. The insert size was assessed using the Agilent Bioanalyzer 2100 system, and qualified insert sizes were accurately quantified using the StepOnePlus™ Real-Time PCR System (valid library concentration > 10 nM). Sequencing libraries were generated using the NEBNext® Ultra™ RNA Library Prep Kit for Illumina® (NEB, U.S.A.) following the manufacturer’s recommendations, and index codes were added to attribute sequences to each sample (GEO number: GSE137418).

### Identification of significant DEGs

DEmRNAs and DElncRNAs were identified by the IDEP website (http://bioinformatics.sdstate.edu/idep/). Genes with false discovery rates (FDRs) ≤ 0.1, |log2FC| ≥ 1.5 and *P*-value ≤ 0.01 were selected as candidate genes. Heatmaps were utilized to identify the gene expression differences between the control group and the 6-TG group. DEmRNAs and DElncRNAs are shown in Supplementary Tables S1 and S2, respectively.

### Kyoto Encyclopedia of Genes and Genomes pathway analysis

Pathway analysis was used to investigate the DEmRNAs according to the Kyoto Encyclopedia of Genes and Genomes (KEGG; http://www.genome.jp/kegg/) database. The volcano plot of GSEA was structured on the website (http://www.webgestalt.org/option.php). Hierarchical clustering and heatmap plot analyses were performed for the ten main down-regulated pathways via the METASCAPE website (https://metascape.org/gp/index.html#/main/step1). The genes in the top ten pathways and seven pathways are shown in Supplementary Tables S3 and S4, respectively.

### Protein–protein interaction network submodule analysis

The protein–protein interaction (PPI) network of genes involved in the main pathways was constructed to understand the relationships of different genes. The PPI network was constructed using Cytoscape3.7.2. The submodule of the PPI network was analyzed by using the MCODE plugin of Cytoscape. GO and KEGG pathway enrichment analyses for the DEGs involved in the module were performed.

### Collection of mRNA–lncRNA–miRNA data

DEmRNAs in the MCODEs were collected for further analysis. Then, miRNAs of mRNAs were predicted from StarBase (http://starbase.sysu.edu.cn/) and miRTarBase (http://mirtarbase.mbc.nctu.edu.tw/). Correlation analysis was performed to evaluate the interaction between DEmRNAs and DElncRNAs. Pairs with absolute values of Pearson correlation coefficients not less than 0.95 were selected. The miRNAs of these lncRNAs were predicted by DIANA tools (http://carolina.imis.athena-innovation.gr/diana_tools/web/index.php).

### Construction of ceRNA network

According to the ceRNA hypothesis, there were potential interactions among lncRNAs, mRNAs and miRNAs. We constructed the ceRNA coregulated network of DElncRNAs, DEmiRNAs and DEmRNAs using echarts. In brief, we used TarBase and miRTarBase to predict 18 miRNAs of 21 hub DEmRNAs. We also predicted coexpressed lncRNAs of the 21 hub genes according to Pearson correlation coefficients ≥ 0.95. Then, we utilized DIANA tools to predict the coexpressed miRNAs of the abovementioned DElncRNAs. Finally, we constructed a ceRNA network according to Supplementary Table S5 on the echarts website (https://echarts.apache.org/examples/zh/editor.html?c=sankey-simple).

### RNA extraction and qPCR analysis

Total RNA was extracted with TRIzol (15596026, Invitrogen, U.S.A.) reagent following the manufacturer’s instructions. Complementary DNA (cDNA) was synthesized using TransScript All-in-One First-Strand cDNA Synthesis SuperMix for qPCR (One-Step Gdna Removal) (AT341, TRAN, China). qPCR was performed with FastStart Essential DNA Green Master (06924204001, Roche, U.S.A.) via a StepOnePlus Real-Time PCR system. Primers sequences are listed in Supplementary Table S6. The results were analyzed using the 2^−ΔΔ*C*_T_^ method. The qPCRs were all repeated three times.

### Survival analysis

To evaluate the potential roles of genes in the ceRNA network in the patients, we used Cox regression analysis of survival packages and Kaplan–Meier curves on OncoLnc (https://www.oncolnc.org) and Kaplan–Meier plotter (http://kmplot.com/analysis/) to perform Kaplan–Meier survival analyses of the association between the expression of DElncRNAs, DEmiRNAs and DEmRNAs in the ceRNA network and the prognosis of patients. We only selected the TNBC sample set, which is composed of 255 patients with ER negative, PR negative, and HER-2 negative.

### Statistical analysis

Data are presented as the means ± SEMs. The experiments were repeated at least two times in triplicate. Statistical analysis was performed using GraphPad Prism 6.01 (GraphPad Software, U.S.A.). **P<0.01 was considered statistically significant.

## Results

### Cell growth was inhibited by 6-TG

**Figure 1:**
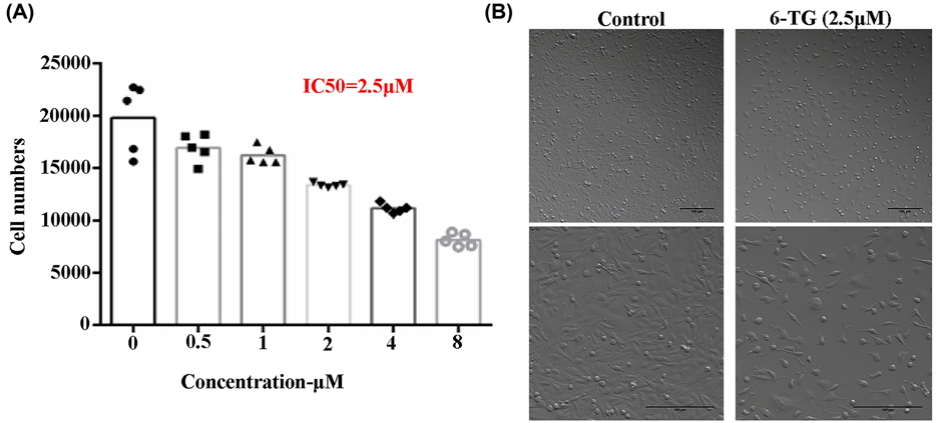
Cell growth was inhibited by 6-TG (**A**) Cells were incubated with different concentrations of 6-TG for 48 h. Cell viability was determined by the CCK-8 assay. IC_50_ values were calculated by GraphPad Prism 6.01 software. (**B**) The morphology of MDA-MB-231 cells in the control group and 6-TG treatment group.

To examine whether 6-TG affects the growth of MDA-MB-231 cells, MDA-MB-231 cells were treated with different concentrations of 6-TG. As shown in Figure 1A, 6-TG revealed significant cytotoxic effects in a dose-dependent manner. The IC_50_ of 6-TG in 48 h was 2.5 μM. Moreover, the cell morphology was changed after 6-TG treatment (Figure 1B); specifically, the cell volume shrank, and intercellular connections were evacuated. Our results indicated that MDA-MB-231 cells were sensitive to 6-TG.

### Identification of DEmRNAs and DElncRNAs and functional enrichment analysis

**Figure 2:**
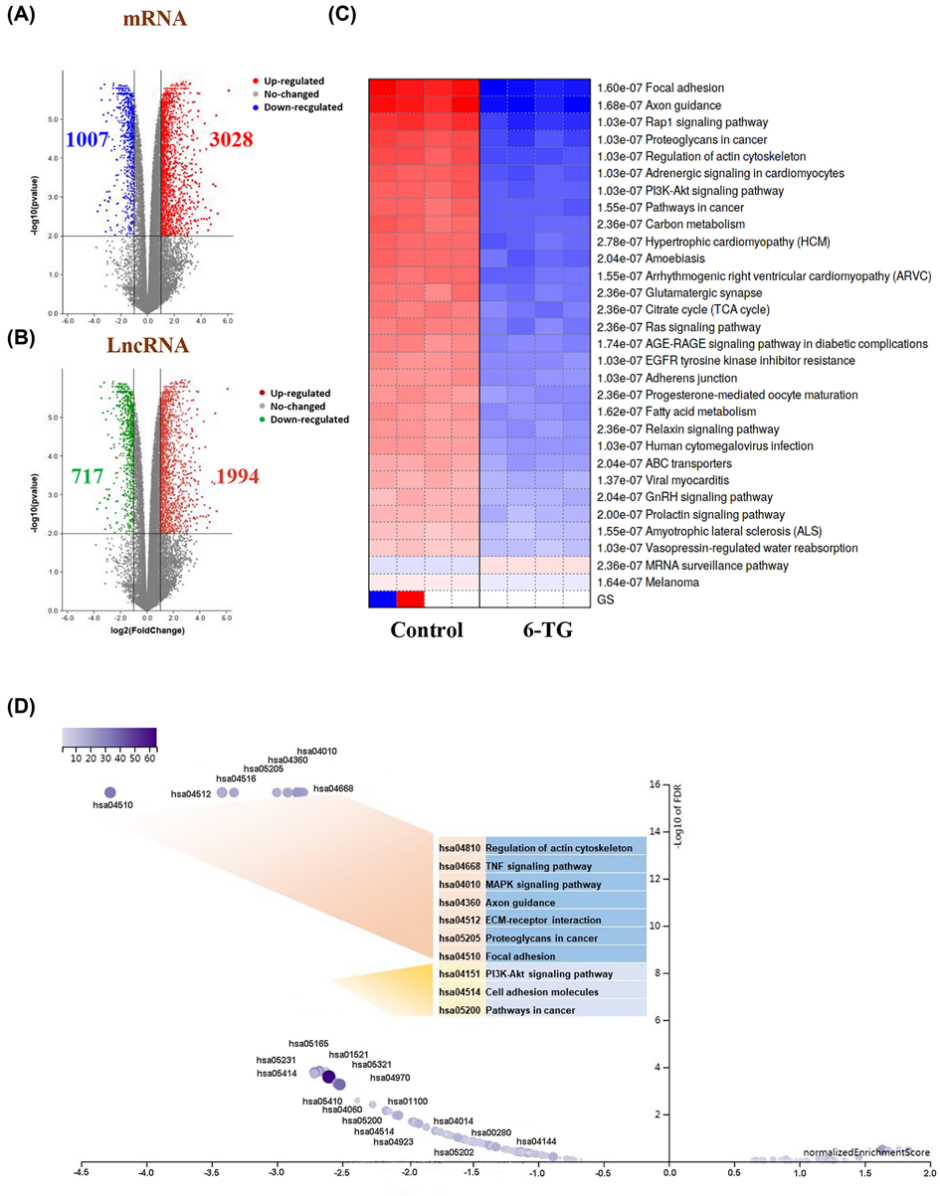
Differentially expressed gene analysis (**A,B**) Volcano plots of differentially expressed mRNAs and lncRNAs. The y-axis represents the negative log (base 10) of the FDR, and the x-axis represents the log (base 2) of FC. Each point represents a gene. The dots in blue or green denote down-regulated DEGs, the dots in red denote up-regulated DEGs, and the dots in black denote non-DEGs. (**C**) PGSEA heatmap of the DEmRNAs. (**D**) Volcano plot of GSEA results based on the KEGG pathway.

To study the gene expression profile of MDA-MB-231 cells under 6-TG treatment, we identified DEmRNAs and DElncRNAs. We found 3028 up-regulated and 1007 down-regulated DEmRNAs (Figure 2A) and 1994 up-regulated and 717 down-regulated DElncRNAs (Figure 2B) after treatment with 6-TG. To determine the biological functions and pathways of the 4035 DEmRNAs, the PGSEA method was performed to analyze KEGG pathway enrichment. As shown in Figure 2C, the MRNA surveillance pathway was the only pathway with up-regulated gene enrichment among the top 30 pathways. Another 29 pathways were enriched for the down-regulated genes. The pathways enriched for down-regulated DEmRNAs were divided into two groups according to the GSEA volcano (Figure 2D). The top ten pathways were regulation of actin cytoskeleton, TNF signaling pathway, MAPK signaling pathway, axon guidance, ECM–receptor interaction, proteoglycans in cancer, focal adhesion and PI3K-Akt signaling pathway, cell adhesion molecules and pathway in cancer. The above results showed that 6-TG mainly inhibited cell proliferation by down-regulating the oncogenic pathway.

### Core PPI network construction

**Figure 3:**
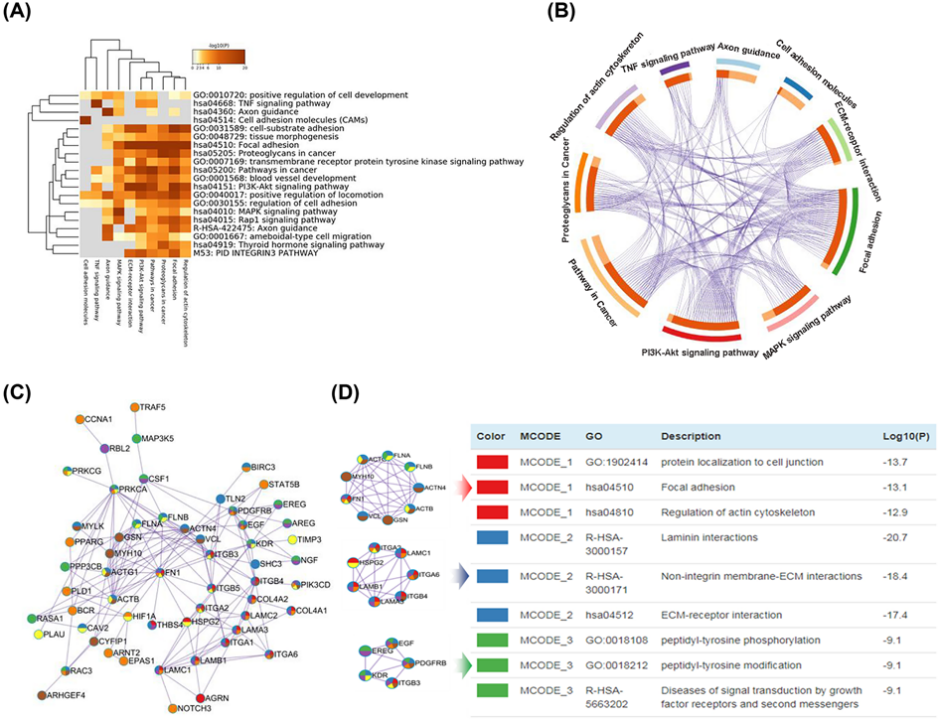
Core PPI network construction (**A**) Heatmap of enriched terms across gene lists of ten pathways was constructed. Enriched clusters are colored by *P*-values. (**B**) The picture indicates the overlap between gene lists. Purple curves link identical genes only at the gene level. The inner circle represents gene lists, where hits are arranged along the arc. Genes that hit multiple lists are colored in dark orange, and genes unique to a list are shown in light orange. (**C**) The PPI network was constructed using the hub genes. Network of enriched terms represented as pie charts, where pies are color-coded based on the identities of the gene lists of each pathway. (**D**) MCODE components identified in the gene lists from the PPI network. All lists merged and colored by counts.

Genes involved in the ten tumor-related pathways mentioned above were selected to perform pathway enrichment based on the Metascape website. The pathway heat map showed that the main functional pathways were: Regulation of actin cytoskeleton, MAPK signaling pathway, ECM–receptor interaction, Proteoglycans in cancer, Focal adhesion, PI3K-Akt signaling pathway and Pathway in cancer (Figure 3A). Moreover, the seven main functional pathways shared most of the genes (Figure 3B). Next, we utilized the genes shared by seven pathways to construct a PPI network to explore the relationship among DEGs by Cytoscape software, as shown in Figure 3C. We determined that the key hub nodes were *FN1, FLNA, FLNB, VCL, GSN, MYH10, ACTG1, ACTN4, KDR* and *EREG.* Meanwhile, MCODE analysis showed that the 21 hub nodes were involved in three subnets, named MCODE-1, MCODE-2 and MCODE-3 (Figure 3D). All the results indicated that the downregulation of the hub genes in the seven pathways played an important role in the inhibition of breast cancer cells under 6-TG treatment.

### Construction of the ceRNA networks

**Figure 4:**
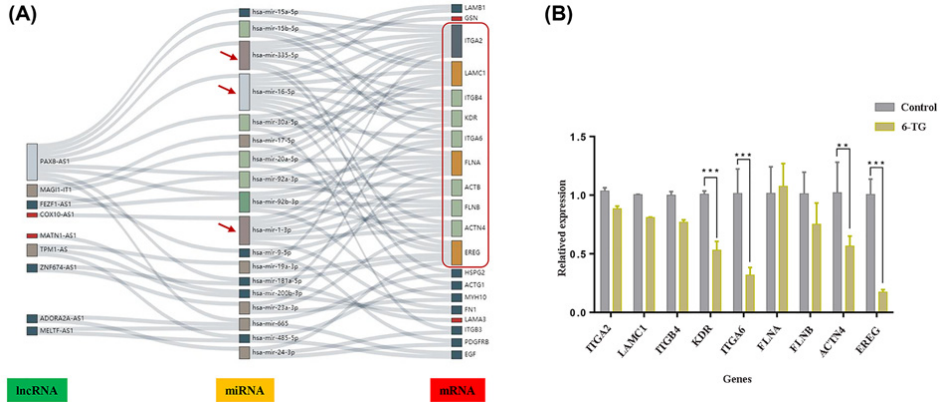
Construction of ceRNA network (**A**) The regulatory network of mRNAs–miRNAs–lncRNAs. The more interactions, the bigger the shapes. (**B**) The qPCR validation of the expression levels of the hub genes (**P<0.01, ***P<0.001).

To better understand the pivotal roles of DEmRNAs and DElncRNAs in DMA-MB-231 cells under 6-TG treatment, a ceRNA network was constructed. We used TarBase and miRTarBase to predict 18 miRNAs according to the abovementioned 21 hub DEmRNAs. We also predicted coexpressed lncRNAs of the 21 hub genes according to Pearson correlation coefficients ≥ 0.95. Then, we utilized DIANA tools to predict the coexpressed miRNAs of the abovementioned DElncRNAs. Echarts was used to construct the coexpression network. As shown in Figure 4A, the coexpression network consisted of 18 miRNAs, 9 DElncRNAs and 20 DEmRNAs. *Hsa-miR-16* (connection degree = 10), *Hsa-miR-1* (connection degree = 7) and *Hsa-miR-335* (connection degree = 6) were the top three miRNAs that targeted most mRNAs, suggesting that they were major miRNAs in the coexpression network. Only six lncRNAs (*PAX8-AS1, MAGL1-AS1, FEIF1-AS1, MATN1-AS1, TPM1-AS1* and *ZNF674-AS1*) were identified in the network according to the Lnc2Cancer 3.0 database. Therefore, *PAX8-AS1* targeted the top two miRNAs and interacted with the majority of mRNAs *ITGA2, LAMC1, ITGB4, KDR, FLNA, ACTN4* and *EREG.* To verify our analysis, the expression levels of the majority of mRNAs were assessed using qPCR. As shown in Figure 4B, the expression levels of *KDR, ITGA6, ACTN4* and *EREG* were significantly lower in the 6-TG group than in the control group. These data suggested that ceRNA played a potential role in regulating gene expression in MDA-MB-231 cells treated with 6-TG.

### Association between key genes and overall survival

**Figure 5:**
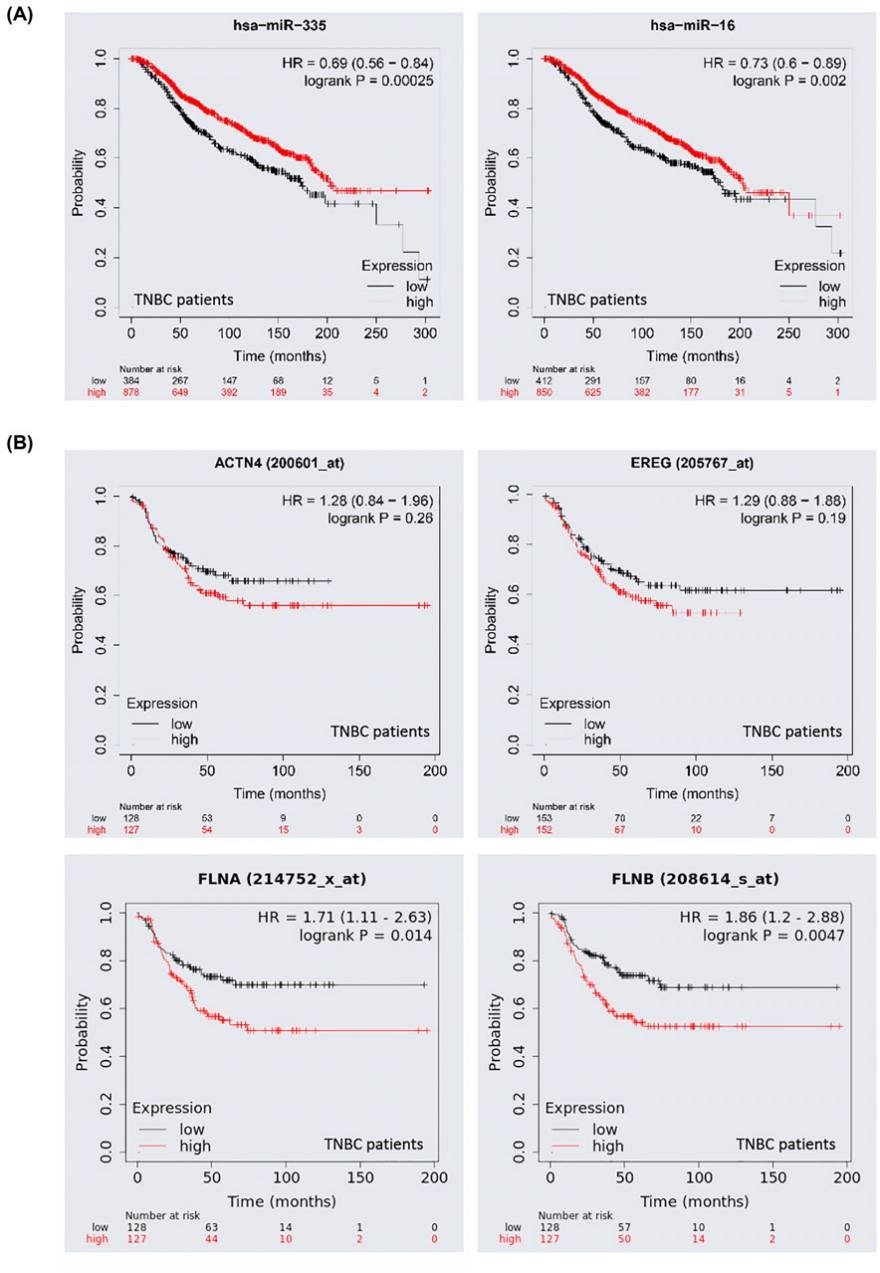
Kaplan–Meier survival curves for the hub genes in the ceRNA network (**A,B**) The survival curves of *hsa-miR-16, hsa-miR-335, ACTN4, EREG, FLNA* and *FLNB*. The TNBC samples with ER negative, PR negative and HER-2 negative were selected in this analysis.

We performed Kaplan–Meier survival analysis for each DEmiRNA and DEmRNA in the constructed ceRNA network to obtain RNAs that exhibited a close relation to the prognosis of patients. As shown in Figure 5A, the miRNAs *Hsa-miR-16* and *Hsa-miR-335* seemed to exhibit protective functions, as the prognosis of patients with higher expression levels was longer than that of patients with lower expression levels. However, the *ACTN4, EREG, FLNA* and *FLNB* mRNAs (Figure 5B) were regarded as risky, and there were negative relationships between their expression and prognosis. These data suggested that 6-TG played a positive role in prolonging the survival time.

## Discussion

TNBC, which exhibits strong invasiveness, rapid recurrence and poor prognosis, is difficult to treat because of the lack of targets. Chemotherapy is the primary established systemic treatment for patients with TNBC [13,14]. 6-TG is a classic chemotherapy drug for leukemia treatment. Several studies have reported the potential treatment role of 6-TG in breast cancer. Kazuhiko et al. [15,16] reported that 6-TG-induced DNA mismatches could activate the MSH2/MLH1-BRCA1-ATR-Chk1 pathway, leading to G_2_ arrest. Our previous study showed that 6-TG induces FAS-mediated exogenous apoptosis and p21-dependent G_2_/M arrest in MCF-7 breast cancer cells by regulating *DNMT1* [8]. However, the regulatory effect of 6-TG on the lncRNA–miRNA–mRNA ceRNA network in the TNBC cell line MDA-MB-231 has not been reported. In the present study, we comprehensively analyzed the ceRNA network in MDA-MB-231 cells treated with 6-TG, aiming at revealing the inhibition mechanism induced by 6-TG in the regulation of lncRNAs in the ceRNA network. Moreover, we screened hub genes to understand the potential treatment of 6-TG in the ceRNA network for TNBC.

To the best of our knowledge, changes in a variety of cancer signaling pathways are involved in the development and progression of breast cancer. Previous studies have shown that MAPK signaling, the PI3K/AKT pathway and focal adhesion pathways play important roles in malignant transformation in TNBC [17,18]. *KDR* (*VEGFR-2*) was reported as a regulator of cell proliferation, and its reduced expression could inhibit cell growth [19]. Yasuhiro et al. [20] reported that *Vash2* inhibited tumor growth by down-regulating *EREG* and *IL11*, suggesting the antitumor effects of *EREG* in tumors. Fortunately, our results showed that the tumor-related pathways, Regulation of actin cytoskeleton, MAPK signaling pathway, ECM–receptor interaction, Proteoglycans in cancer, Focal adhesion, PI3K-Akt signaling pathway and Pathway in cancer, were inhibited by 6-TG. In addition, we identified the hub genes in these pathways, such as *FN1, FLNA, FLNB, ACTN4, VCL, GSN, MYH10, ACTG1, EREG* and *KDR*, suggesting that they were the dominant genes in these pathways under 6-TG treatment.

ceRNAs are transcripts that can regulate each other at the post-transcriptional level by competing for shared miRNAs [21]. Amelia et al. [22] reported that miR-16 could target Bcl2 and induce apoptosis in chronic lymphocytic leukemia. In addition, miR-16 regulates cell cycle progression by targeting *CCND1, CCND3, CCNE1* and *CDK* [23–25]. *MiR-335* suppresses metastasis and migration by targeting the progenitor cell transcription factor *SOX4* and has been identified as a metastasis suppressor miRNA in human breast cancer [26]. An et al. [27] found that the circular RNA circZMYM2 competed with *miR-335-5p* to regulate cell proliferation, apoptosis and invasion in pancreatic cancer. However, we did not observe any studies reporting lncRNAs with potential competing abilities. Increasing evidence indicates the crucial role of lncRNAs in the ceRNA network in terms of modulating other RNA transcripts [28]. Wei et al. [29] reported that thyroid cancer patients with higher *PAX8‐AS1* expression levels had shorter RFS times. While the isoform of *PAX8-AS1* is named *PAX8-AS1-N*, it can bind to *miR-17-5p* and upregulate *miR-17-5p* targets, such as *PTEN, CDKN1A*, and *ZBTB4*. The reduced expression of *PAX8-AS1-N* indicated poor survival of breast cancer patients [30]. Nevertheless, the regulation of *PAX8-AS1* in ceRNAs is unknown. In our results, we found decreased lncRNA *PAX8-AS1* expression under 6-TG treatment in MDA-MB-231 cells. The lncRNA *PAX8-AS1* targeted the top two miRNAs and interacted with the majority of mRNAs in the ceRNA network. Moreover, the mRNAs with reduced expression after 6-TG treatment were consistent with those predicted by *PAX8-AS1*. Therefore, we predicted that *PAX8-AS1* may sponge *miR-16* and *miR-335*. This lncRNA can mediate *ACTN4, EREG, KDR, FLNA*, and *FLNB* expression by competing with *miR- 16* and *miR-335* to participate in the progression of MDA-MB-231 cells. Therefore, lncRNA *PAX8-AS1* may inhibit MDA-MB-231 cells by sponging *miR-16* and *miR-335* to exert ceRNA regulation. Meanwhile, the high expression levels of *miR-16* and *miR-335* and the low expression levels of *ACTN4, ERGE, FLNA*, and *FLNB* could prolong patient survival, suggesting that 6-TG influenced the gene expression pattern and played a positive role in TNBC treatment.

In summary, we showed that *PAX8-AS1* is the main lncRNA influenced by 6-TG and that *PAX8-AS1* regulates hub genes in tumor pathways by competitively binding with *miR-16-5p* and *miR-335-5p*. The present study contributes to the exploration of the ceRNA regulatory mechanism of 6-TG and provides valuable insight for further functional research.

## Supplementary Material

Supplementary Tables S1-S6

## Data Availability

The datasets generated for the present study can be found under the GEO number: GSE137418.

## Abbreviations

CCK-8: Cell Counting Kit-8
ceRNA: competitive endogenous ribonucleic acid
DElncRNA: differentially expressed lncRNA
DEmRNA: differentially expressed mRNA
DMSO: dimethyl sulfoxide
ER: estrogen receptor
FDR: false discovery rate
GEO: Gene Expression Omnibus
HER-2: human epidermal growth factor receptor 2
KEGG: Kyoto Encyclopedia of Genes and Genomes
lncRNA: long noncoding RNA
miRNA: microRNA
ncRNA: noncoding RNA
PPI: protein–protein interaction
PR: progesterone receptor
TNBC: triple-negative breast cancer
6-TG: thioguanine
