# Supplementary material for: The regulatory effect of 6-TG on lncRNA–miRNA–mRNA ceRNA network in triple-negative breast cancer cell line
Source: Biosci Rep. 2021 Feb 3;41(2):BSR20203890. doi: 10.1042/BSR20203890 (PMC7859320; doi:10.1042/BSR20203890)
Supplement: Supplementary Tables S1-S6 [file BSR-2020-3890_supp.zip › BSR-2020-3890_suppST6.docx]

**Table S6:** **Primers sequences.**

| **Gene** | **Forward primer (5’ to 3’)** | **Reverse primer (5’ to 3’)** |
| --- | --- | --- |
| ITGA2 | CCTACAATGTTGGTCTCCCAGA | AGTAACCAGTTGCCTTTTGGATT |
| LAMC1 | GGCAACGTGGCCTTTTCTAC | AGTGGCAGTTACCCATTCCTG |
| ITGB4 | GCAGCTTCCAAATCACAGAGG | CCAGATCATCGGACATGGAGTT |
| KDR | GTGATCGGAAATGACACTGGAG | CATGTTGGTCACTAACAGAAGCA |
| ITGA6 | ATGCACGCGGATCGAGTTT | TTCCTGCTTCGTATTAACATGCT |
| FLNA | GGAGGAGGCAAAAGTGACCG | ACTTATCCACGTACACCTCGAAG |
| FLNB | GTGAACAAACGCATCGGCAA | ACCAGACCCAAGATGAGCTTC |
| ACTN4 | TCGGGGCAGAAGAGATTGTG | CTGGATGGCGAACCTAAGGA |
| EREG | GTGATTCCATCATGTATCCCAGG | GCCATTCATGTCAGAGCTACACT |
